# A35 ENDOSCOPIC SUBMUCOSAL DISSECTION (ESD) OUTCOMES IN GASTROINTESTINAL SUBMUCOSAL TUMORS (SMT): A RETROSPECTIVE STUDY

**DOI:** 10.1093/jcag/gwae059.035

**Published:** 2025-02-10

**Authors:** M Youssef, C Ching Hui Yee, R Bechara

**Affiliations:** University of Toronto, Toronto, ON, Canada; Division of Internal Medicine, McGill University Faculty of Medicine and Health Sciences, Montreal, QC, Canada; Kingston Health Sciences Centre, Kingston, ON, Canada

## Abstract

**Background:**

Gastrointestinal submucosal tumors (SMTs) range from benign entities to malignant tumors that require surveillence or resection. The role of ESD in management of SMTs remains unclear.

**Aims:**

We aim to evaluate the clinical and technical outcomes of ESD in the management of SMTs.

**Methods:**

This study included patients with SMTs who underwent ESD at the Kingston Health Sciences Center (KHSC) between May 2017 to September 2024. Data was collected on patient demographics and tumor characteristics including size, pathology, differentiation, depth, and lymphovascular invasion (LVI). Technical outcomes were analyzed with respect to procedure time (min), technical efficiency (min/cm^2^), en-bloc and R0 resection rates. En-bloc resection was defined as resection of the lesion piecemeal and R0 indicated negative margins on microscopic examination. We also reported clinical outcomes including length of stay, perioperative adverse events (e.g. bleeding or perforation), strictures, and tumor recurrence rates at the time of follow-up. Descriptive analysis was used to summarize the data.

**Results:**

This case series included 22 patients (7 males, 15 females) with SMTs (6 esophageal, 16 gastric). 40.1% of patients (9/22) had endoscopic biopsy prior to ESD. The median specimen area was 3.8 cm^2^ (min-max range 0.38-18.2 cm^2^). Procedure times ranged between 5-138 mins with a mean time of 49.6 mins (SD= 33.1 mins) and mean efficiency of 13.7 min/cm^2^. 100% of patients had en-bloc resections and 86.3% (19/22) had R0 resections. There were no perioperative adverse events and most patients (21/22) were discharged the same day with the exception of one patient who stayed for 6 days postoperatively for (chronic) pain management. The pathology of the six esophageal tumors showed 3 leiomyomas, 2 Granular Cell Tumors (GCT), and 1 lipoma. Of the sixteen gastric lesions, 6 were Gastrointestinal Stromal Tumors (GIST), 6 were neuroendocrine tumors (NET), 1 leiomyoma, 1 lipoma, 1 inflammatory fibroid polyp, and 1 heterotopic pancreatic tissue. In both GIST and NET tumors, 5/6 lesions were Grade 1 (well-differentiated) and 1/6 was Grade 2 (moderately differentiated). The depth of the lesions was into the submucosa and there was no lymphovascular invasion (LVI). With regards to the esophageal tumors that required endoscopic follow-up (n= 4), the mean follow-up time was 8.5 months and there was no tumor recurrence. Of the gastric lesions that required endoscopic follow-up (n= 14), the mean follow-up time was 26.2 months and there was no tumor recurrence. There were no strictures observed on endoscopic follow-up for any of the lesions.

**Conclusions:**

ESD for SMTs is a safe procedure with high en-bloc and R0 resection rates, along with low recurrence rates on follow-up. Larger scale studies are needed to further explore the efficacy and safety of ESD in the management of SMTs.

**Funding Agencies:**

None

